# Insecticidal and sterilizing effect of Olyset Duo^®^, a permethrin and pyriproxyfen mixture net against pyrethroid-susceptible and -resistant strains of *Anopheles gambiae s.s.*: a release-recapture assay in experimental huts

**DOI:** 10.1051/parasite/2015027

**Published:** 2015-10-21

**Authors:** Armel Djènontin, Ludovic P. Ahoua Alou, Alphonsine Koffi, Barnabas Zogo, Elves Duarte, Raphael N’Guessan, Nicolas Moiroux, Cédric Pennetier

**Affiliations:** 1 Faculté des Sciences et Techniques-Université d’Abomey-Calavi Cotonou Benin; 2 Institut de Recherche pour le Développement (IRD), Maladies Infectieuses et Vecteurs, Ecologie, Génétique, Evolution et Contrôle (MIVEGEC), UM-CNRS 5290 IRD 224 Cotonou Benin; 3 Centre de Recherche Entomologique de Cotonou (CREC) Cotonou Benin; 4 Institut Pierre Richet (IPR), Institut National de Santé Publique (INSP) Bouaké Côte d’Ivoire; 5 London School of Hygiene and Tropical Medicine Keppel street London UK; 6 IRD MIVEGEC, UM-CNRS 5290 IRD 224 Montpellier France

**Keywords:** *Anopheles gambiae s.s*, Pyrethroid, Resistance, Bed net, Insect growth regulator

## Abstract

In the context of the widespread distribution of pyrethroid resistance among malaria vectors, we did a release-recapture trial in experimental huts to investigate the insecticidal and sterilizing effects of a novel long-lasting net (LN), Olyset^®^ Duo, incorporating a mixture of permethrin (PER) and the insect growth regulator (IGR), pyri-proxyfen (PPF). An LN containing PPF alone and a classic Olyset^®^ Net were tested in parallel as positive controls. The effect of progressive number of holes (6, 30, or 150) that may accrue in nets over time was simulated. We used two laboratory *Anopheles gambiae s.s.* strains: the susceptible Kisumu strain and the pyrethroid-resistant VK-Per strain having solely *kdr* as resistance mechanism. The effect of these nets on the reproductive success of blood-fed females that survived the different LNs conditions was recorded. Regardless of the mosquito strain, the LNs containing PPF alone with as many as 30 holes drastically reduced the number of eggs laid by females succeeding in feeding, i.e. fecundity by 98% and egg hatching rate (fertility) by 93% relative to untreated control net. Very few of the resistant females blood fed and survived under the Olyset^®^ Duo with similar number of holes (up to 30) but of these few, the inhibition of reproductive success was 100%. There was no evidence that the Olyset^®^ Duo LN with 150 holes impacted fecundity or fertility of the resistant colony. The efficacy of Olyset^®^ Duo is encouraging and clearly illustrates that this new net might be a promising tool for malaria transmission control and resistance management.

## Abstract (fr)

Dans le contexte d’expansion de la résistance aux pyréthrinoïdes chez les vecteurs de paludisme, nous avons réalisé une expérience de lâcher-recapture en cases expérimentales pour étudier l’efficacité insecticide et stérilisante d’une nouvelle moustiquaire à longue durée d’action imprégnée d’un mélange de perméthrine (un pyréthrinoïde) et de pyriproxyfen (PPF, un inhibiteur de croissance des insectes). Des moustiquaires imprégnées de PPF seul et des Olyset^®^ Net standard ont servi de témoins positifs. L’expérience a été réalisée sur des moustiquaires comportant un nombre variable de trous (6, 30 ou 150) pour simuler différents niveaux d’usure. Les moustiques testés étaient les souches de laboratoire d’*Anopheles gambiae s.s.* sensible (Kisumu) et résistante (VK-Per), qui possède uniquement la mutation kdr comme mécanisme de résistance. Les capacités reproductives des femelles gorgées ayant survécu au contact de ces différentes moustiquaires ont été mesurées. Quelle que soit la souche de moustique, seules les moustiquaires imprégnées de PPF avec jusqu’à 30 trous ont significativement réduit le nombre d’œufs pondus par les femelles (-98% de fécondité et -93% de fertilité) comparativement aux moustiquaires non-traitées. Peu de femelles résistantes ont survécu au contact avec l’Olyset^®^ Duo avec jusqu’à 30 trous et aucune d’entre elles n’a pondu d’œufs. En revanche, nous n’avons observé aucun effet sur la fécondité et la fertilité des femelles ayant survécu au contact avec l’Olyset^®^ Duo trouée 150 fois. L’efficacité d’Olyset^®^ Duo est encourageante et montre clairement que cette nouvelle moustiquaire pourrait être un outil prometteur pour le contrôle de la transmission du paludisme et de la gestion de la résistance.

## Introduction

During the last decade, expansion of financial support and significant scale-up of malaria control tools have led to a large reduction in malaria incidence and/or mortality [30]. Despite this major reduction of the burden, malaria is still a major public health concern, with an estimated 207 million cases and 627,000 deaths that occurred in 2012 [30].

Moreover, the rapid and widespread resistance to insecticides and antimalarial drugs represents a serious threat to sustainability of current malaria control tools [2, 17, 29, 30]. Major mechanisms of insecticide resistance involve either an alteration in the rate of insecticide detoxification (metabolic mechanisms) and/or mutations within the target site of the insecticide. There two main mechanisms confer phenotypic resistance to the four insecticide families used for malaria vector control (carbamates, organophosphates, organochlorines, and pyrethroids) [10, 27]. The pyrethroid insecticides are the only class deployed for bed net impregnation because they are highly efficacious, fast-acting against insects at low doses, and have acceptably low toxicity to mammals [32]. The development of alternative tools against resistant mosquitoes is therefore an urgent need.

One strategy to prevent or delay the development of resistance is to use at least two insecticides having unrelated modes of action in combination on the same bed net. Many mixtures and combinations have been studied in the last decade with the objective of impregnating bed nets for use in the future [3, 11, 18, 22, 24, 25]. Among these, the currently available combination products are long-lasting insecticidal treated nets (LNs) impregnated with pyrethroid and synergist, e.g. piperonyl butoxide (PBO). These LNs were developed to specifically target malaria vectors bearing metabolic resistance mechanisms, with encouraging results [4, 16, 23, 28].

Another candidate for combining with insecticides for vector control is an insect growth regulator (IGR), e.g. pyriproxyfen (PPF), that impedes insect morphogenesis, embryogenesis, and reproduction [5]. Due to its unique mode of action that sterilizes adult malaria vectors and the lack of reported resistance to PPF in mosquitoes, PPF could complement the insecticidal arsenal to better control malaria transmission [9]. In this context, Sumitomo Chemical^©^ developed a new LN impregnated with both permethrin (PER) and PPF. This new product showed promising performances in the laboratory (Rossignol M, pers. comm.) and against one wild *An. gambiae* population [19]. Here, its performance was investigated against both susceptible and pyrethroid-resistant laboratory strains of *An. gambiae* through a release-recapture experiment in experimental huts in Benin. The aim of this trial was to measure the efficacy of the new LN incorporating both PER and PPF in comparison with nets impregnated with PER or PPF alone (as positive controls) and untreated net (as negative control). The impact of these nets on the reproductive success of susceptible and resistant *An. gambiae* was of interest and therefore investigated when accrued numbers of holes appear in them in the field.

## Materials and methods

### Study area

The study was carried out in four experimental huts situated in Akron (6°30′ N and 2°47′ E), a village on the periphery of Porto Novo, the administrative capital of Benin. The site is a horticultural area covering approximately 20 ha.

### Mosquito strains

Two strains of *Anopheles gambiae s.s.* were used:

- Kisumu is a standard susceptible strain originating from Kenya, maintained for many years in the laboratory and free of any detectable insecticide resistance mechanisms (VectorBase, http://www.vectorbase.org, KISUMU1). The susceptibility of Kisumu mosquitoes to insecticides is checked monthly using WHO test kit and the absence of resistance mechanisms was also checked by PCR and biochemical assays.
- VK-Per originated from Burkina Faso and was exposed to constant permethrin selections in the laboratory until becoming homozygous for the *kdr* mutation (L1014F). The fixed status of the *kdr* mutation in this strain is checked monthly. VK-Per strain displayed the same expression level of metabolic resistance enzyme as Kisumu [17].

### Design of huts

The huts are made from concrete bricks, with a corrugated iron roof, a ceiling of thick polyethylene sheeting, and a concrete base surrounded by a water-filled channel to prevent entry of ants. Mosquito access is *via* four window slits constructed from pieces of metal, fixed at an angle to create a funnel with a 1 cm wide gap. Mosquitoes fly upward to enter through the gap and downwards to exit; this precludes or greatly limits exodus through the aperture enabling the majority of entering mosquitoes to be accounted for. A single veranda trap made of polyethylene sheeting and screening mesh measuring 2 m long, 1.5 m wide, and 1.5 m high, projects from the back wall of each hut. Movement of mosquitoes between hut and veranda is unimpeded during the night.

### Study design

The following treatments were tested in huts:

- Untreated polyethylene net;
- Olyset^®^ Net, a permethrin 2% (w/w) incorporated into polyethylene net;
- Pyriproxyfen (PPF) 1% (w/w) incorporated into polyethylene net;
- Olyset^®^ Duo, a permethrin 2% (w/w) + pyriproxyfen 1% (w/w) incorporated into polyethylene net.

Before testing in the experimental huts, the nets (including control) were deliberately holed with three different holing conditions to mimic possible wear and tear under natural condition of use:

- 6 holes of 4 cm × 4 cm as per WHO guidelines, corresponding to 0.07% of the surface of a family size Olyset^®^ Net, distributed proportionally on each side of the net;
- 30 holes of 4 cm × 4 cm corresponding to 0.36% of the surface of a family size Olyset^®^ Net, distributed proportionally on each side of the net;
- 150 holes of 4 cm × 4 cm corresponding to 1.83% of the surface of a family size Olyset^®^ Net, distributed proportionally on each side of the net.

In order to even out the surface content of active ingredient on the different types of nets, nets were washed three times to remove initial content according to the WHOPES standard washing methods and active ingredient left to regenerate over one week prior to the release-recapture trial in huts [31].

For each treatment and each holing condition, four release sessions (50 mosquitoes each) of *An. gambiae* Kisumu and four of VK-Per strains were run in each hut, respectively, allowing a complete rotation of treatments in the huts.

### Volunteer participants’ recruitment and mosquito collections

Adult volunteers recruited among inhabitants of villages close to the field site signed a written informed consent form. Their vaccination status against yellow fever was checked before enrolment and they were medically followed up throughout the study. Sleepers were rotated among huts and treatments each night of the study following a Latin square design. They entered into the hut at 21:00 and remained inside until dawn. The Ethics Committee of the Health Ministry of Benin approved the protocol for the study.

All window slits were blocked to prevent entry/exit of any free wild mosquitoes. Batches of 50 unfed mosquito females 3–5 days old were released in the evening (21:00) when sleepers got under the net. In the morning (06:00), the sleeper collected dead mosquitoes from the floor of the hut, the veranda traps, and inside the nets. Resting mosquitoes were collected using glass tubes from inside the net, on the walls, and ceiling of the hut and exit trap. Mosquitoes were scored by location as dead or alive and as fed or unfed. Live mosquitoes were placed in small cups with access to honey solution for 24 h to assess delayed mortality.

The primary outcomes measured were [31]:

- induced exophily (the proportion of mosquitoes found in the veranda relative to the total collected mosquitoes);
- blood-feeding inhibition (the reduction in blood feeding of mosquitoes in the treatment hut relative to the control huts);
- immediate and delayed mortality (the proportion of dead mosquitoes at the time of collection and those dying after 24 h).

All outcomes were calculated as the proportions of recaptured mosquitoes.

### Fecundity and fertility assessment

Living blood-fed females collected from all compartments of the huts were counted and brought back to the laboratory. For each treatment, blood-fed females were put by batch of up to 20 females in cardboard cups (450 mL). We previously filled the bottom of the cardboard cups with a 1 cm high layer of wetted cotton, covered with a filter paper disk to allow females to lay their eggs. The females in cardboard cups were maintained with honey solution in an observation room at 28 °C and 80% RH for 5 days. After 5 days, we checked the mortality of females in cups. We took a picture of each filter paper to count the eggs using Egg Counter software [15]. All eggs from the control and treated batches were immersed in water. After 6 days, the number of larvae (3rd and 4th instars) was checked to determine the hatching rates. Efficacy was measured in terms of fecundity (number of eggs laid per female) and fertility (% of eggs that hatched).

### Statistical analysis

The software “R” was used for the statistical analyses [26]. The proportions of mosquitoes that attempted to exit were killed within the hut and had blood fed successfully, were analyzed using a logistic regression model. The “brglm” function from the brglm package was used for the analysis [13]. It allowed the fitting of binomial-response regression models using the bias-reduction method developed by Firth [7]. These procedures return estimates with improved frequentist properties (bias, mean squared error) that are always finite, even in cases where the maximum likelihood estimates are infinite (data separation).

Count data related to the actual number of eggs laid by batches of surviving females and those hatching (i.e. number of larvae per surviving female) were analyzed using negative binomial regression. Differences in mortality and KD rates between the treatments from the WHO cone bioassays were compared using the χ^2^ test.

## Results

### Insecticidal efficacy of treatments

The trial was carried out between June 21, 2012 and August 2, 2012. Four release-recapture replicates were run for each holing configuration and each strain, leading to a total of 4800 mosquitoes released. Of these, 1883 *An. gambiae s.s.* Kisumu and 2087 *An. gambiae s.s.* VK-Per were recaptured. The results for the treatment-induced exophily, blood-feeding inhibition, and induced mortality are summarized in Table 1 for LNs condition with 6, 30, and 150 holes.

**Table 1.** Summary results of the release-recapture of *An. gambiae s.s.* (Kisumu and VK-Per strains) relative to the treatments and holing configuration.

Treatments			*N* females caught	Exophily			Blood feeding			Mortality		
				Inside veranda	Rate (%)	IE (%)	Blood-fed	Rate (%)	BFI (%)	Dead	Rate (%)	Corrected rate (%)
Kisumu	Control	6 holes	152	45	29.6^a,c^	–	54	35.5^a^	–	3	2.0^a^	–
		30 holes	149	31	20.8^a,b^	–	77	51.7^b^	–	3	2.0^a^	–
		150 holes	161	29	18.0^b,f^	–	140	87.0^c,h^	–	1	0.6^a^	–
	Pyriproxyfen	6 holes	181	67	37.0^c^	NS	72	39.8^a,j^	NS	3	1.7^a^	−0.3
		30 holes	148	29	19.6^b,f^	NS	86	58.1^b^	NS	5	3.4^a^	1.4
		150 holes	153	44	28.8^a,c^	59.7	132	86.3^c^	NS	1	0.7^a^	0.0
	Olyset	6 holes	146	5	3.4^d^	−88.4	13	8.9^d^	74.9	146	100^b^	100
		30 holes	156	6	3.9^d,e^	−81.5	17	10.9^d,e^	78.9	156	100^b^	100
		150 holes	160	9	5.6^d^	−68.8	30	18.8^e^	78.4	159	99.4^b^	99.4
	Olyset Duo	6 holes	156	14	9.0^d^	−69.7	0	0^f^	100	156	100^b^	100
		30 holes	159	8	5.0^d^	−75.8	5	3.1^d,f^	93.9	159	100^b^	100
		150 holes	162	3	1.9^e^	−89.7	12	7.4^d^	91.5	162	100^b^	100
VK-Per	Control	6 holes	180	21	11.7^b^	−	152	84.4^c^	–	3	1.7^a^	–
		30 holes	173	16	9.3^d^	–	160	92.5^h^	–	1	0.6^a^	–
		150 holes	181	42	23.2^a,b^	–	178	98.3^i^	–	1	0.6^a^	–
	Pyriproxyfen	6 holes	181	25	13.8^b^	NS	146	80.7^c,k^	NS	4	2.2^a^	0.6
		30 holes	172	20	11.6^b^	NS	148	86.1^c,h^	NS	3	1.7^a^	1.2
		150 holes	180	38	21.1^a,f^	NS	179	99.4^i^	NS	3	1.7^a^	1.1
	Olyset	6 holes	173	34	19.7^b,f^	68.5	95	54.9^b^	35.0	95	54.9^c^	54.2
		30 holes	166	24	14.5^b^	NS	101	60.8^b^	34.2	136	81.9^d^	81.8
		150 holes	175	90	51.4^g^	121.6	144	82.3^c^	16.3	92	52.6^c^	52.3
	Olyset Duo	6 holes	171	28	16.4^b,f^	NS	32	18.7^e^	77.8	149	87.1^d,e^	86.9
		30 holes	165	20	12.1^b^	NS	72	43.6^j^	52.8	152	92.1^e^	92.1
		150 holes	170	61	35.9^c^	54.6	123	72.4^k^	26.4	123	72.4^f^	72.2

The numbers in the same column sharing the letter superscript do not differ significantly (*p* ≥ 0.05).

#### Control

The proportions of the resistant VK-Per mosquitoes that blood fed under the untreated net (84–98%) were significantly higher than with the Kisumu strain (36–87%) (*p* < 0.001). The blood-feeding rates increased with the number of holes. The mortality rates in the control hut were universally low (<3%), regardless of the strain or the number of holes, indicating that there was no contamination or carryover of active ingredient from one hut to another during the trial.

#### PPF-treated net

Regardless of the hole configurations and the mosquito strains, PPF-LN alone induced less than 4% mortality. Blood feeding of Kisumu strain ranged from 40% (6 holes) to 86% (150 holes) and that of VK-Per ranged from 80% (6 holes) to 100% (150 holes). The induced exophily was not significantly different from the control treatment except for Kisumu strain with 150 holes.

#### Olyset^®^ Net

Blood-feeding rates were highly inhibited with the Kisumu strain (74–79%) and inhibited to a lesser extent with the VK-Per strain (16–34%). Mortality rates corrected for control were nearly 100% with Kisumu and significantly lower with VK-Per mosquitoes (51–82%). With Olyset^®^ Net, fewer Kisumu mosquitoes were collected in the veranda trap compared to the control hut, while significantly more VK-Per mosquitoes were collected in the veranda trap with Olyset^®^ Net with 6 holes and 150 holes (*p* < 0.05).

#### Olyset^®^ Duo

Olyset^®^ Duo strongly inhibited blood-feeding rates of the Kisumu strain by 79–100%, regardless of the number of holes. With both strains, the BFI rates for Olyset^®^ Duo were significantly higher than those of the Olyset^®^ Net (*p* < 0.05) although within each hole category. The impact on Kisumu (79–100%) was greater than for VK-Per (27–87%). BFI decreased with the number of holes in the nets. The decrease was more pronounced with the VK-Per than the Kisumu strain: between 79% and 91% of Kisumu were still prevented from blood-feeding under the LNs with 150 holes compared to only 16–27% for VK-Per under the same type and condition of LNs. Olyset^®^ Duo killed 100% of Kisumu mosquitoes whereas it killed between 73% and 92% of VK-Per. Overall, Olyset^®^ Duo killed significantly greater proportions of the VK-Per mosquitoes than did Olyset^®^ Net (*p* < 0.05).

### Fecundity and fertility of surviving blood-fed females

During the study of fecundity and fertility, we scored 51,790 eggs laid by 1682 surviving blood-fed females collected from the release-recaptures and 41,328 derived larvae. The results of fecundity and fertility relative to the LN products are summarized in Table 2 for LNs conditions with 6, 30, and 150 holes.

**Table 2.** Summary results of fecundity and fertility among Kisumu and VK-Per mosquitoes exposed to the various treatments.

	Treatment	Number of holes	Survival		*N* eggs laid	Fecundity	Fertility		IGR effect
			*N* surviving blood-fed female	% surviving blood-fed female rate		Mean number of eggs/female	(*N*) Number of larvae	Hatching rate (%)	Mean number of larvae/female
Kisumu	Control	6	51	33.6^a^	2452	49^a^	1914	78.1^a^	38^a^
		30	74	49.7^b^	5425	71^a^	3990	73.6^b^	52^a,d^
		150	140	87.0^c^	7016	51^a^	5593	79.7^a^	40^a^
	Pyriproxyfen	6	72	39.8^a^	148	2^b^	47	31.7^c^	0^b^
		30	81	54.7^b^	7	0^b^	0	0.0^d^	0^b^
		150	132	86.3^c^	34	0^b^	19	55.9^b^	0^b^
	Olyset Net	6	0	0^d^	NA	NA	NA	NA	NA
		30	0	0^d^	NA	NA	NA	NA	NA
		150	0	0^d^	NA	NA	NA	NA	NA
	Olyset Duo	6	0	0^d^	NA	NA	NA	NA	NA
		30	0	0^d^	NA	NA	NA	NA	NA
		150	0	0^d^	NA	NA	NA	NA	NA
VK-Per	Control	6	151	83.9^c^	8155	54^a^	6802	83.4^e^	45^a^
		30	159	91.9^c,e^	8715	55^a^	7623	87.5^f^	48^a,d^
		150	177	97.8^e^	9854	56^a^	7654	77.7^a^	43^a^
	Pyriproxyfen	6	145	80.1^c^	18	0^b^	1	5.6^g^	0^b^
		30	147	85.5^c^	2	0^b^	0	0.0^d^	0^b^
		150	176	97.8^e^	479	3^b^	295	61.6^b^	2^b^
	Olyset Net	6	37	21.4^f,a^	999	29^c^	800	80.1^a,e^	23^a,e^
		30	20	12.1^f^	940	49^c,a^	700	74.5^b^	36^a^
		150	76	43.4^b,a^	5593	78^d^	4422	79.1^a,e^	61^c^
	Olyset Duo	6	6	3.5^d^	0	0^b^	NA	NA	0^b^
		30	6	3.6^d^	0	0^b^	NA	NA	0^b^
		150	43	25.2^a^	1953	39^c,a^	1468	75.2^a,b^	29^a,e^

The numbers in the same column sharing the letter superscript do not differ significantly (*p* ≥ 0.05) according to the logistic regression. NS: Not significant.

Firstly, the proportions of surviving blood-fed females were analyzed. Both the Olyset^®^ Net and Olyset^®^ Duo killed all blood-fed Kisumu females and drastically decreased survivorship of blood-fed VK-Per females (4–12% survival) compared to the control (92%) (*p* < 0.001). There was no significant difference in survivorship between the PPF LN and the control. The highest proportions of live blood-fed females were observed in huts with LNs having 150 holes (43% for the Olyset^®^ Net and 25% for Olyset^®^ Duo).

Analysis of fecundity in live blood-fed Kisumu females showed that the PPF-LN reduced the mean number of eggs per female by >98% (*p* < 0.001).

The Olyset^®^ Net never impacted the mean number of eggs per live VK-Per female (*p* > 0.05). None of the very few (6–7 blood-fed VK‐Per *Anopheles*) that survived the Olyset^®^ Duo with 6 and 30 holes laid eggs, leading to 100% fecundity reduction. More VK-Per females blood-fed and survived the Olyset^®^ Duo LN with 150 holes but with this level of holes there was not a significant effect on the mean number of eggs produced per female compared to the control net with the same number of holes (*p* > 0.05) (Table 2).

The analysis of the fertility of the eggs laid by blood-fed females that survived showed that PPF LN alone significantly reduced the hatching rate for both Kisumu and VK-Per strains, regardless of the number of holes. There was no clear evidence that the Olyset^®^ Duo significantly impacted the hatching rate of mosquitoes (*p* > 0.05).

The overall IGR effect is illustrated by the mean number of larvae obtained per surviving blood-fed female (Table 2). The PPF LN reduced the mean number of larvae per Kisumu or VK-Per female by about 99% (*p* < 0.001), regardless of the mosquito strain or the number of holes. Olyset^®^ Net did not influence the mean number of larvae per VK-Per females (*p* > 0.05). With Olyset^®^ Duo containing 6 and 30 holes, the number of larvae per VK-Per females was zero because none of the few females that blood-fed and survived laid eggs. By contrast, the Olyset^®^ Duo with 150 holes did not reduce the mean number of larvae per VK-Per female compared to the control net with 150 holes (*p* > 0.05).

## Discussion

The present study investigated the insecticidal and sterilizing efficacy of the new product, Olyset^®^ Duo, on both susceptible and *kdr*-resistant laboratory *An. gambiae s.s.* mosquitoes in comparison with LNs impregnated with permethrin (Olyset^®^ Net) or PPF alone.

Against the susceptible *An. gambiae s.s.* Kisumu strain, both Olyset^®^ Duo and Olyset^®^ Net killed 100% of exposed mosquitoes. By contrast, a reasonable number of *kdr*-resistant *An. gambiae s.s.* mosquitoes survived exposure to these LNs, confirming that *Kdr* mutation by itself confers some level of phenotypic resistance. Differences in behavior close to the bed net might induce a variable contact time with the impregnated surface. It is interesting to note that the killing effect of permethrin increased when it was used in combination with pyriproxyfen on Olyset^®^ Duo relative to the Olyset^®^ Net for VK-Per. Two hypotheses might explain this pattern: either there is a difference in the bleed rate of permethrin in the combination LN relative to the Olyset^®^ Net [19] or an additive/synergistic interaction between permethrin and pyriproxyfen, although no evidence of such an interaction is available in the literature [8, 14]. It is also possible that the presence of PPF on Olyset^®^ Duo interacts with the irritancy and/or repellence property of permethrin, thereby allowing mosquitoes to rest for longer thus picking up higher doses than Olyset^®^ Net alone.

The results clearly showed that pyriproxyfen LN alone reduced the mean number of eggs per blood-fed females. These findings confirmed the results of a previous study demonstrating fecundity and fertility reduction in adult mosquitoes exposed to bed nets treated with PPF [1, 20, 21]. We showed that Olyset^®^ Duo with a number of holes as high as 30 still greatly reduced the mean number of eggs (fecundity) of blood-fed pyrethroid-resistant *An. gambiae s.s.* that survived this LN. It is worth noting that when hole number increased to 150, Olyset^®^ Duo failed to have an impact on fecundity, although this number of holes in PPF-treated net did reduce the mean number of eggs per VK-Per female. This suggests that the irritant effect of permethrin limited the contact between the female mosquitoes and the treated surface, leading to reduced exposure to pyriproxyfen and thereby limited impact on fecundity. The good efficacy of Olyset^®^ Duo with 6 or 30 holes to sterilize females was encouraging and clearly illustrated that this new LN might be a promising tool to control pyrethroid-resistant *An. gambiae s.s.* and has potential for resistance management. Nevertheless, 5 days after exposure (the end of the period given to lay eggs), only 6 out of 22 and 6 out of 13 VK-Per females collected in the experimental hut with the Olyset^®^ Duo LN with 6 and 30 holes respectively, were alive. It would be important to replicate the trial to strengthen our results. Moreover, it is also crucial to study the efficacy of such new tools against pyrethroid-resistant mosquitoes bearing other resistance mechanisms such as metabolic alone and combined with the *kdr* mutation.

The limitation of this study was the number of surviving females. We suggest to use the LN with 30 holes (instead of WHOPES-standard six holes) to investigate the efficacy of Olyset^®^ Duo against multi-resistant mosquitoes, in order to increase the number of blood-fed females that survive the Duo net without decreasing the time of contact with the LN too significantly (as suggested by the results with Olyset^®^ Duo with 150 holes). In order to better design future experiments, it would also be very useful to study the relationship between tarsal contact time and pyriproxyfen effects as the rationale behind the overall technology of Olyset^®^ Duo is to be able to sterilize pyrethroid-resistant mosquitoes that probe through pyrethroid-treated net and survive, to possibly transmit malaria elsewhere. In adult mosquitoes, the period of exposure to pyriproxyfen in relation to mosquito blood meals affects the mosquitoes’ ability to produce viable offspring [12]. A recent study showed that only female mosquitoes that blood-fed one day prior to pyriproxyfen exposure produced no viable offspring during that gonotrophic cycle [9]. This requires an unlikely scenario in which mosquitoes would rest more than 24 h on bed nets after feeding. In the present study, released mosquitoes were probably in contact with the pyriproxyfen-treated bed net before their blood meal and just after it. The use of PPF as a complementary vector control tool on mosquito resting places might also be an interesting opportunity. Deploying IRS with pyriproxyfen to complement the protective effect of LN within homes might also be an attractive alternative strategy to explore within the framework of managing insecticide resistance in malaria vectors [6].

We conclude that the new Olyset^®^ Duo LN mixing permethrin and pyriproxyfen showed promising results in terms of protective and sterilizing effects against both susceptible and *Kdr*-resistant *An. gambiae s.s.* The next step is to study its efficacy against wild multi-resistant *An. gambiae s.s.* populations from different ecological settings before implementing a larger-scale study with epidemiological outputs.

## Conflict of interest

This work was supported financially by Sumitomo Chemical. Funders participated in the study design and the decision to publish, but they have no role in data collection, analysis, and preparation of the manuscript. The authors declare that they have no competing interests.
